# Mixed Reality Technology to Deliver Psychological Interventions to Adolescents With Asthma: Qualitative Study Using the Theoretical Framework of Acceptability

**DOI:** 10.2196/34629

**Published:** 2023-07-26

**Authors:** Kelsey Sharrad, Caitlin Martini, Andrew Tai, Nicola Spurrier, Ross Smith, Adrian Esterman, Ian Gwilt, Debra Sandford, Kristin Carson-Chahhoud

**Affiliations:** 1 Translational Medicine and Technology Group Australian Centre for Precision Health University of South Australia Adelaide Australia; 2 Department of Respiratory and Sleep Medicine Women's and Children's Hospital Adelaide Australia; 3 Robinson Research Institute University of Adelaide Adelaide Australia; 4 Department of Health and Ageing Government of South Australia Adelaide Australia; 5 Australian Research Centre for Interactive and Virtual Environments University of South Australia Adelaide Australia; 6 UniSA Clinical and Health Sciences University of South Australia Adelaide Australia; 7 UniSA Creative University of South Australia Adelaide Australia; 8 Health and Medical Sciences Faculty University of Adelaide Adelaide Australia; 9 Adelaide Medical School University of Adelaide Adelaide Australia; 10 South Australian Health and Medical Research Institute Adelaide Australia

**Keywords:** asthma, augmented reality, virtual reality, mixed reality, psychological distress, adolescent, cognitive behavioral therapies, mental health

## Abstract

**Background:**

Interactive, mixed reality technologies such as augmented reality, virtual reality, and holographic technology may provide a novel solution to fast-track the translation of evidence into practice. They may also help overcome barriers to both mental health and asthma management service uptake, such as cost, availability of appointments, fear of judgment, and quality of care.

**Objective:**

This study aimed to investigate if mixed reality technology is an acceptable mechanism for the delivery of a component of cognitive and behavioral therapies for the management of elevated psychological distress among young people with asthma.

**Methods:**

To explore the perceived acceptability of these technologies, mixed reality tools were evaluated via qualitative, 1-on-1 interviews with young people with asthma and symptoms of psychological distress, parents/caregivers of young people with asthma and symptoms of psychological distress, and relevant health professionals. The Theoretical Framework of Acceptability was used for the deductive coding of the recorded interview transcripts.

**Results:**

This study enrolled the following participants: (1) 3 adolescents with asthma and symptoms of psychological distress with a mean age of 14 (SD 1.7) years; (2) 4 parents/caregivers of adolescents with asthma with a mean age of 55 (SD 14.6) years; and (3) 6 health professionals with a mean age of 40.8 (SD 4.3) years. A total of 4 constructs—experienced affective attitude, experienced effectiveness, self-efficacy, and intervention coherence—were coded in all participant transcripts. The most frequently coded constructs were experienced affective attitude and intervention coherence, which were reported a total of 96 times. The least frequently coded construct was anticipated opportunity cost, which was reported a total of 5 times. Participants were mostly positive about the mixed reality resources. However, some concerns were raised regarding ethicality, particularly regarding privacy, accessibility, and messaging. Participants noted the need for technology to be used in conjunction with face-to-face engagement with health professionals and that some patients would respond to this type of delivery mechanism better than others.

**Conclusions:**

These results suggest that mixed reality technology to deliver psychological interventions may be an acceptable addition to current health care practices for young people with asthma and symptoms of psychological distress.

**Trial Registration:**

Australia and New Zealand Clinical Trials Registry ACTRN12620001109998; https://anzctr.org.au/Trial/Registration/TrialReview.aspx?id=380427

## Introduction

### Background

Australia has one of the highest asthma prevalence rates in the world [1], with 11% of people being affected, as per self-report [2]. Asthma is the leading cause of disease burden among young Australians aged 5 to 14 years, with 460,000 (10%) young people affected [2]. 

According to a 2018 report, people with asthma were more likely to experience psychological distress (the experience of symptoms of anxiety and depression at subclinical levels [3]) than individuals without asthma (15% vs 8.7% for high levels and 11% vs 3.4% for very high levels, respectively) [4]. In adolescents, a 2014 survey of 533 Australians between 12 and 25 years of age found that half the adolescents with asthma experienced symptoms of heightened psychological distress [5]. The most common causes of distress were similar to those of adolescents without asthma; however, asthma-related problems also contributed to psychological distress in this sample. The study highlights that psychological distress is not uncommon and has both asthma and nonasthma–related triggers.

### Psychological Interventions for People With Asthma

Due to the bidirectional relationship between asthma and symptoms of psychological distress [6], psychological interventions may offer techniques and strategies to manage both psychological distress and symptoms of asthma, thus reducing the risk of exacerbations [7]. Research in this population is limited, with a 2005 systematic review (currently being updated [8]) unable to draw conclusions about the potential role of psychological interventions for children with asthma due to heterogeneous data [9]. Cognitive and behavioral therapy (CBT) is a type of psychological intervention that helps patients recognize and modify thoughts and behaviors that may be detrimental to their health and well-being [10]. CBT-based strategies could be useful in the treatment of symptoms of heightened psychological distress in people with asthma; however, evidence suggests that engagement with treatment is low in this population, with reports estimating that 4 (80%) out of 5 children and adolescents who could potentially benefit from psychological intervention are reportedly not accessing it [11]. In a 2016 Australian survey, 48% of parents of adolescents aged between 12 and 17 years reported that their child refused help; however, 39% of parents were not sure where to get help, 33% could not afford help, and 29% reported that they could not get an appointment [12]. Even when services are accessed, a recent study conducted in 21 countries found that only 9.8% of individuals with an anxiety disorder received possibly adequate treatment based on evidence-backed guidelines [13].

### Technology for Health Interventions

A recent systematic review of 28 studies found that text-based internet searches were the most commonly identified help-seeking approach among adolescents, along with other internet communities [14]. Reasons for this preference for help-seeking via the internet include anonymity and privacy, immediacy, ease of use, inclusivity, connection with others, and an increased sense of control [14]. However, the effectiveness and safety of unguided self-help vary significantly due to the wide range and quality of sources available [10]. Evidence-based psychological interventions delivered via technologies such as smartphone apps and online resources—also known as e-psychology, eHealth, or e-mental health interventions—have the potential to be an effective option for psychological well-being support and may increase access to and quality of care [15]. A 2016 meta-analysis exploring the use of digital CBT in children and adolescents reported reduced anxiety in the intervention group compared with the control. No statistically significant differences in efficacy were observed between digital and in-person treatment modalities [16]. 

### Mixed Reality for Health Interventions

Interactive technologies such as augmented reality (AR), virtual reality (VR), and holographic technology (also known as mixed reality technology) may provide a novel solution to aid in the timely translation of evidence-based treatment into practice. Using a smartphone as a viewing device, AR superimposes digital information into the real world so that content seems to coexist with reality [17]. VR requires a headset to view content, allowing the viewer to feel completely immersed in the digital world. On the other hand, holographic technology uses the projection of diffracted light to create images. Mixed reality technologies deliver treatment and health care information through videos, graphics, and animation, which can address low health literacy [17,18], allow for tailoring for individual population characteristics (eg, age, language), increase engagement [19], increase accessibility of information [20], and enable real-time updates of content, thus reducing the evidence-to-practice gap. AR and VR have been studied in multiple health care contexts, including cancer, autism, and chronic pain, with feasibility, acceptability, and early efficacy data suggesting that these modes of intervention delivery may be beneficial for adolescents [21-23].

In children and adolescents with asthma, a recent scoping review found that digital health interventions (including VR and AR interventions) were a promising option for asthma management and treatment delivery and were perceived positively by health care professionals and patients [24]. Methodologically rigorous research is needed to ensure that evidence-based, easily accessible digital interventions are made available [24,25].

### Study Aims

Accordingly, this study aimed to investigate whether mixed reality technology is an acceptable mechanism for the delivery of a component of CBT to manage symptoms of elevated psychological distress among young people with asthma.

## Methods

### Primary Methodology

**Figure 1:**
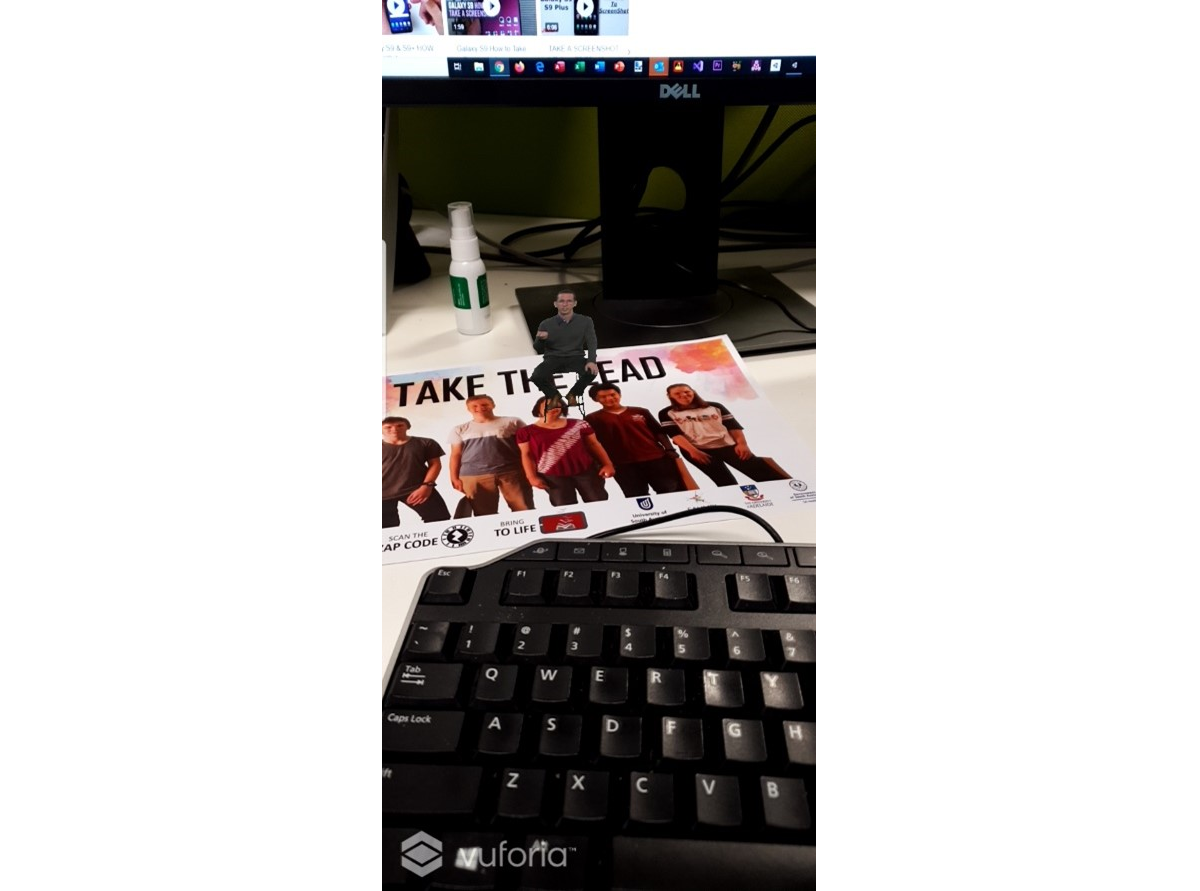
This screenshot of the augmented reality (AR) technology shows a member of the research team sitting atop the paper resource. The AR has been activated by the smartphone camera.

**Figure 2:**
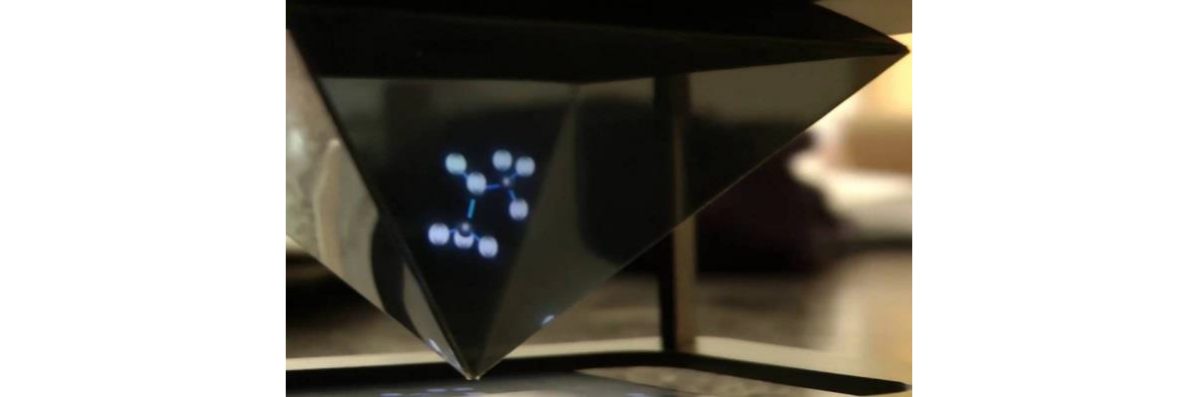
The plastic projector that sits atop a smartphone to create the holographic projection.

This paper focuses on the acceptability of mixed reality technology as a delivery mechanism for a component of CBT to manage symptoms of elevated psychological distress among young people with asthma. The mixed reality tools utilized for this qualitative study were preexisting resources, including 1 bespoke AR resource (Figure 1), 1 VR resource, and 1 holographic resource (Figure 2). For the AR resource, participants used a smartphone camera to make a digital image of a human body appear as though it were in real space (Figure 1). The digital image provides information about asthma and the effect it can have on various body systems, demonstrating how psychoeducation (a technique used in CBT) could be offered using this technology. For the VR resource, participants wore a cardboard headset over their eyes, into which a smartphone was placed. The smartphone played a VR-specific video of calming nature landscapes while a meditative audio track played. The holographic resources involved a small plastic projector (Figure 2) being placed over a smartphone. When a specific video was played, images appeared in 3D within the plastic projector. The VR and holographic resources demonstrate the capacity of these technologies to deliver mindfulness strategies. Three different technologies were chosen for hypothesis-generating purposes to establish if 1 mechanism is more acceptable than others.

To evaluate perceptions of usability and appropriateness, 1-on-1 interviews were carried out with the target audience. Semistructured moderator guides (Multimedia Appendices 1-3) were developed to direct interviews with adolescents with asthma, parents/caregivers of adolescents with asthma, and health professionals, including general pediatricians, psychologists, psychiatrists, nursing staff, and pediatric respiratory specialists. Participants were not provided with access to the mixed reality resources before the interview. During the interviews, the research staff conducted a brief training session, and participants were provided with the mixed reality resources with which to interact in their own time. Interviews ran for approximately 1 hour and took place in meeting spaces in the respiratory department at 2 large teaching hospitals in metropolitan Adelaide, South Australia, or online via Zoom software. Sessions were audio recorded, and verbatim transcripts were sent back to the participant for validation after the interview. Participants also completed a simple questionnaire (Multimedia Appendices 4-6) requesting demographic information, as well as self-reported measures of asthma knowledge and technology useability that will be explored in subsequent reports.

### Sampling/Recruitment

#### Overview

Participants were all identified through a pediatric respiratory specialist at the Women’s and Children’s Hospital. Health professionals were recruited through word of mouth. Purposive sampling was utilized to ensure a good representation of participant characteristics to meet the requirements of the research question.

Hospital staff provided potential participants with a copy of the participant information sheet and consent form for their review. If the participants were interested in learning more, consent to contact them was obtained, and the research staff was given contact details to follow up for screening and consenting procedures.

#### Inclusion Criteria for Young People

Young people were eligible for inclusion in this study if they (1) were aged between 13 and 17 years, (2) were formally diagnosed with asthma by a health professional (inpatients or outpatients), (2) had experienced or were currently experiencing symptoms of psychological distress determined by the Kessler Psychological Distress Scale (K10+) [26], (3) had access to a smartphone with the owner's permission to use it during the interview, and (4) were English speaking or able to understand written English.

#### Inclusion Criteria for Parents/Caregivers

Participants were eligible for inclusion in this study if they (1) were the parent/ caregiver of an adolescent with asthma (aged 13 to 17 years) who currently had or had reported in the past elevated symptoms of psychological distress (did not need to be a child actively participating in this study), (2) had access to a smartphone and can use smartphone technology (basic level), and (3) were English speaking or able to understand written English.

#### Inclusion Criteria for Health Professionals

Health professionals were eligible for inclusion in this study if they (1) had been practicing in their respective fields for at least 12 months, (2) had access to a smartphone and could use smartphone technology (basic level), and (2) were English speaking and able to understand written English.

#### Exclusion Criteria for All Participant Groups

Participants with an intellectual disability or cognitive impairment that would inhibit their ability to provide informed consent and participate in the study were ineligible to participate. Young people with a history of epilepsy or other contraindications for the use of VR were also ineligible to participate.

### Qualitative Data Analysis

Qualitative data were coded using three prespecified lenses to enable insight into different aspects of the mixed reality interventions: (1) the Theoretical Domains Framework (TDF)[2], the Theoretical Framework of Acceptability (TFA), and (3) the Enlight protocol. This paper will focus solely on data obtained through the TFA, while the TDF and Enlight protocols will be featured in separate reports. Deductive thematic coding was used with a framework analysis technique based on the TFA [27]. The TFA comprises 7 constructs reflecting the multifaceted nature of acceptability, incorporating both anticipated and experienced thoughts, beliefs, and feelings regarding the intervention [27,28]. The 7 constructs are ethicality, self-efficacy, intervention coherence, affective attitude, burden, opportunity costs, and perceived effectiveness, with the last 4 separated into “anticipated” and “experienced” subcategories. The TFA was shown to be successful in exploring acceptability in health promotion interventions [29]. Previous research demonstrates a more robust understanding of acceptability when a framework is applied compared with no framework [27-29].

All transcripts were coded by 2 independent researchers (authors KS and CM), with discrepancies resolved through consensus or discussion with a third party (author KCC). During the coding process, quotes were determined to be generally positive, negative, or neutral toward the mixed reality technology. A standardized pilot-tested data extraction Microsoft Excel (Microsoft Corp) template was used for data management. 

### Ethics Approval

This study was conducted following the principles of the Declaration of Helsinki [30] and received ethical approval from the Human Research Ethics Committee for the Women's and Children's Health Network (HREC/18/WCHN/172) and the University of South Australia Ethics Committee (201967).

## Results

### Participants

A total of 19 participants were approached to take part in the study, with 13 completing interviews. Two young people and 1 parent withdrew from the project for psychological reasons, and another 2 young people and 1 parent were considered ineligible. Interviews were conducted by 1 of 3 researchers (authors KS, ZK, and KCC).

The 13 participants included 3 (23%) adolescents with asthma, 4 (31%) parents of young people with asthma, and 6 (46%) health professionals, including 2 (33%) psychologists, 1 (16%) psychiatrist, 1 (16%) medical consultant, 1 (16%) respiratory nurse consultant, and 1 (16%) respiratory sleep nurse consultant. The average duration of experience among health professionals was 17.08 (SD 5.87) years. As per the inclusion criteria, all adolescents reported to have experienced or were currently experiencing symptoms of psychological distress determined by the K10+ scale. The technology experience level of participants varied greatly. Full demographic details are presented in Table 1.

**Table 1:** Participant demographic data.

Participants		Young people with asthma (n=3)	Caregivers of young people with asthma (n=4)	Health professionals (n=6)
Age (years), mean (SD)		14 (1.7)	55 (14.6)	41 (4.3)
Female sex, n (%)		2 (66)	1 (25)	4 (66)
**Nationality, n (%)**				
	Australia	3 (100)	4 (100)	5 (83)
	United Kingdom	N/A^a^	N/A	1^b^ (17)

^a^N/A: not applicable.

^b^Participant was of dual UK and Australian nationality.

### Qualitative Analysis

#### Overview

A total of 4 constructs, namely, experienced affective attitude, experienced effectiveness, self-efficacy, and intervention coherence, were coded in all 13 participant transcripts. The most frequently coded constructs were experienced effective attitude and intervention coherence, which were reported a total of 96 times, while the least frequently coded construct was anticipated opportunity cost, which was reported a total of 5 times. The remaining categories were coded between 11 and 85 times. Example quotes for each TFA construct are included in Multimedia Appendix 7.

Participant groups were similar in their proportion of positive, negative, and neutral quotes across all constructs (Table 2).

**Table 2:** Positive, negative, and neutral quotes obtained from participant interviews and organized by participant group.

Participant group	Positive, n (%)	Neutral, n (%)	Negative, n (%)
Health professionals	138 (56)	78 (32)	29 (12)
Young people with asthma	48 (64)	23 (31)	4 (5)
Parents of young people with asthma	79 (66)	32 (27)	8 (7)

#### Affective Attitude

Affective attitude is defined as “how an individual feels about taking part in an intervention” [27]. Transcripts were coded with quotes before and after exposure to the intervention to assess anticipated and experienced affective attitudes. There were 25 quotes identified for anticipated affective attitude, with most (n=13, 52%) being positive. Most quotes were identified in interviews with health professionals, with a common theme emerging of the potential for technology to be a useful tool when used in conjunction with a face-to-face consultation with physicians:

I think on its own, I would be worried, but if they're also seeing a therapist, counselor, psychologist...or had a trusted adult and health professional to talk to, I think it could be very positive.

Health professional #6

Young people were also generally positive about the idea of technology:

Um, yeah, definitely. It would help. A lot because you know a lot of people spend most of their time on their phones nowadays, having an app like that. Well, you know, it would probably help a lot and you would be more, you know, you would probably use it more.

Young person #7

Similarly, 96 quotes were identified for experienced affective attitude, with the majority (n=73, 76%) being positive. “Calming,” “cool,” and “engaging” were terms often used by participants in all groups to describe their experience. However, not all experiences were positive. One health professional suggested:

Uh, I probably couldn't be bothered doing it myself, I guess. Um, I think sometimes it's just about grabbing people's attention and to me, I felt that had a sense that young people would say, yeah, yeah, whatever.

Health professional #5

The comment that the holographic technology did not meet expectations commonly occurred among health professionals and parents of young people, along with disappointment about the size of the hologram. As 1 health professional stated:

I think if you kept, like, if you kept it, that kind of size, I think you’d lose engagement. Cause it would just be too little. But if you could have that, so it was, like, a really big thing then that’d be cool.

Health professional #2

#### Perceived Effectiveness

Effectiveness refers to “the extent to which the intervention is perceived as likely to achieve its purpose” [27]. Once again, transcripts were coded to assess both anticipated and experienced effectiveness. A total of 23 quotes were identified for anticipated effectiveness, among which 11 (48%) were positive, 2 (9%) were negative, and 9 (39%) were neutral. When asked if this kind of technology could be useful for young people with asthma, young people tended to answer in the affirmative, with 1 participant explaining:

Um, yeah, definitely. It would help. A lot because you know a lot of people spend most of their time on their phones nowadays, having an app like that. Well, you know, it would probably help a lot and you would be more, you know, you would probably use it more.

Young person #7

As with affective attitude, quotes coded for this domain in transcripts with health professionals focused on the need for technology to be used in conjunction with face-to-face engagement with health professionals. Participants also noted that some people would respond well to information delivered via technology, and others would not. One health professional said:

I think it's a really good adjunct because there are some people that will be, like, perfect for… And there are some people that it will be okay with and there'll be some people that won't engage with it or won't have the actual technology resources themselves, like the phone and the iPads...have access to do it. So, I think it just adds another string to your bow, it's some other way that will work. And it's about figuring out that goodness of fit.

Health professional #4

A total of 85 quotes were coded for experienced effectiveness, among which 56 (66%) were positive, 8 (35%) were negative, and 21 (25%) were neutral. Participants felt that the technologies would be beneficial, particularly for education, given the engaging visual nature of the information. As 1 parent of a young person with asthma stated:

So, the goal is to understand as best you can, what's happening either inside your own body or inside your child's body. Okay. So that's, that's the aim. What is the best mechanism for doing that? Well, to see it really, isn't it? And so then to see it in the most representative and the most real and the most, um, sort of engaging way becomes I guess the goal.

Parent of young person #2

Once again, participants in all groups suggested that some people would respond more positively than others. A young person with asthma said:

It would be very beneficial for them. At least, um, or at least some people, depending on if they prefer it this way or that way. It would be very beneficial to the people who absolutely prefer something like this to learn and to discover more about themselves.

Young person #3

Participants also noted the ease of accessibility of the information. One health professional stated:

Yeah, definitely because that's something that they can access at home and it's something that they can access at any time. Um, it's creating that, um, autonomy to the, to the patient, to the family, um, re…reinforcing education that they might want, but in their own time and privacy of their own home.

Health professional #3

#### Ethicality

Ethicality is “the extent to which the intervention has good fit with an individual’s value system” [27]. A total of 15 quotes were coded for this construct, with the majority (n=8, 53%) being classified as neutral, 2 (13%) as positive, and 5 (33%) as negative. Of these quotes, 12 (80%) were coded in interviews conducted with health professionals, and none were from interviews with young people. Issues identified included questions of accessibility:

They might not have access to reliable internet, or they may not have the money or access to a device, then, you know, it’s all very well having that, but they might not have the ability to use it.

Health professional #1

The issues identified also included questions of privacy:

Um, I think I have concerns over privacy and access to data and so on, in particular as most, um, servers seem to be based overseas.

Health professional #1

Participants were also concerned with the accuracy of online messaging (“*as long as you…stick to your mainstream stuff and um, organizations that publish things*”) and the importance of human interaction:

I think that it’s important that not everything is self-diagnosed and then self-referred to sort of technology treatments, I suppose. I think probably having an element of, um, human interaction is important.

Health professional #5

#### Opportunity Costs

Opportunity costs refer to “the extent to which benefits, profits, or values must be given up to engage in the intervention” [27]. Transcripts were coded to assess both anticipated and experienced effectiveness. A total of 5 quotes were coded for anticipated opportunity costs, all from interviews with health professionals. Health professionals were asked if they thought that learning to use this kind of technology would be a good use of their time; 4 (80%) quotes were positive, and 1 (20%) was negative. The positive comments were replying to the question in the affirmative (*“Yeah. Yes. Because then it would help me to keep up with the young people and know what they're talking about”*), while the negative comment expressed that the technology would not be relevant to her work (*“Not for me. No.”*)

In total, 11 quotes were coded for experienced opportunity costs, with quotes identified from all 3 participant categories of participant. Among these, 8 (73%) were positive, and 1 (9%) was neutral. Health professionals commented on whether learning to use and recommend the technology would be a good use of their time:

Yeah, definitely because that's something that they can access at home, and it's something that they can access at any time.

Health professional #3

They also commented on the accessibility of the technology for intervention delivery:

Particularly if it's, say, if it's an app you download on your phone, if it's a card box that you can easily access, think what people are looking for is that convenience, just download the app. That's sort of a low-cost, no-cost app or the little cardboard boxes. And that again in my sort of world in education. They're easy recommendations to make.

Health professional #4

Likewise, young people with asthma and parents of young people with asthma commented predominantly on the accessibility of the technology. One parent noted that the accessibility would depend on the skills and abilities of the user:

Um, I would say that if it's on the phone, it's more accessible, probably quicker, but that would also depend on your knowledge [inaudible] accessing it.

Parent of young person #6

#### Burden

This construct was also separated into anticipated and experienced subcategories, and it is defined as “the perceived amount of effort that is required to participate in the intervention” [27]. A total of 14 quotes were identified for anticipated burden, with 6 (43%) being positive, 1 (7%) being negative, and 7 (50%) being neutral. Most comments about anticipated burden related to the age of the participants, with comments identified in interviews with all 3 groups of participants. When asked if they thought the technologies would be difficult to use, a health professional said, *“For me? Yes. For a young person? No*.” One parent of a young person agreed:

Probably difficult for my age group. Um, because you know, we didn't grow up with them, but the kids it's just like, they don't find it difficult at all.

Parent of young person #5

Young people with asthma also echoed that sentiment (*“Um, in some cases, maybe*”), while another parent of a young person with asthma disagreed:

I think the awareness of technology these days of people my age as well as the younger generation is pretty reasonable. So, most things you pick up on pretty quickly.

Parent of young person #2

Similarly, some health professionals expressed confidence in their ability to use the technology:

Um, no. I think everyone's pretty up to date relatively with smartphones and that these days, so... no, not really difficult.

Health professional #3

However, another expressed concerns:

I've noticed that I'm getting to an age now where I might not be quite up to speed with all, all the technology. So, you know, that can be a barrier as well if the clinicians and the treating teams aren't up to speed.

Health professional #3

A total of 46 quotes were coded for experienced burden, with the majority (n=30, 65%) being positive. Additionally, 7 (15%) comments were negative, and 5 (11%) were neutral. Many participants used the words “easy” and “simple” to describe their experience using the technologies, expressing that it would be easy to use once familiar with the technologies. As 1 parent of a young person with asthma asserted, *“Um, I suppose anything like that is easy once you're using it for a while.*” Once again, age was cited as a confounding factor, with a young person with asthma saying, *“[It would be] probably easy for young people, and it'd be harder for older people.”* Notably, participants expressed concerns about the burden of holographic technology more than the AR and VR tools. One parent of a young person with asthma said:

I think we'd have to say it was moderately difficult. Right? If I didn't have a skilled proponent of the technology…I would have seen nothing cause you, but you could imagine a little arrangement that has a black bit of cardboard at the back and then something that sort of positions that somehow, need a little bit of thought…and kind of got to position it from above, don't you, otherwise you interrupt one of your pictures.

Parent of young person #2

#### Self-efficacy

This construct refers to “the participant’s confidence that they can perform the behavior(s) required to participate in the intervention” [27]. A total of 29 quotes were coded for this construct, with 17 (59%) being positive, 5 (17%) being negative, and 6 (21%) being neutral. Similarly, in the quotes identified for the burden construct, participants often shared that they found the technology easy. Once again, a few of the adult participants commented that they might find the technology difficult but that a younger person probably would not. One health professional explained *“I’m not tech savvy*,” and 1 parent shared that while some people of his age would adapt well to technology, he found it more difficult:

Look, uh, certainly there would be a certain amount in my generation. I would think that um, possibly, a little bit more, uh... You know, adaptable to computers and things. There's some pretty smart people out there of my age that, you know, it's. Computers and that, and technology is natural for them. It's never been sort of part of what I've ever done if you know what I mean?

Parent of young person #6

Likewise, when the young people with asthma were asked if they would use the tools themselves, most answered in the affirmative, and most indicated that they were comfortable using the technology:

Yes of course I would. These like, really work, very informative and very, very calming.

Young person #7

#### Intervention Coherence

Intervention coherence is defined as “the extent to which the participant understands the intervention and how it works” [27]. There were 96 quotes coded for this construct. While some were unsure about the technology before experiencing the intervention (*“I have heard of it. I don't sort of understand it too much”*), most demonstrated understanding afterward. We also considered that quotes about how the technology could be used outside of this project demonstrated an understanding of the intervention and how it worked and thus were appropriate to this construct. For example, 1 health professional said:

Yeah. To be used as, like, a treatment tool for those kids? I guess it depends on what you're actually wanting to deliver, but I can definitely see [the] potential that if you're giving, um, if you're giving education on how like lungs work and how the pathophysiology of it all and the effectiveness and how medications work like um, your bronchodilators or whatever, then that could be really good cause I can actually see what's happening inside of them. So, I think that would then help them kind of put the picture together as opposed to just talking to them. From an anxiety and depression point of view. Um, yeah, I mean, I think that like a guided meditation type thing, like you did with the [VR] one that could, that could be useful in that kind of scenario.

Health professional #2

## Discussion

### Principal Findings

The findings of this study suggest that mixed reality technologies are generally acceptable to adolescents with asthma, parents of adolescents with asthma, and health professionals. Participants across all 3 groups largely felt positively toward the mixed reality technology (Table 2) and considered the technology to be potentially effective and easy to use. This is also seen in a recent study in hospitalized children and adolescents with chronic illnesses, which found that, when compared to face-to-face CBT, a VR treatment involving education, breathwork, and mindfulness techniques received higher perceived efficacy scores [22]. Similarly, a 2020 study [23] exploring the feasibility and acceptability of a VR intervention for psychological well-being in children and adolescents with cancer found that the technology was viewed positively by health professionals, parents/caregivers, and patients. The largely positive affective attitude toward the mixed reality resources is promising, as affective attitude is an important determinant of behavior [31] and has been demonstrated to be a predictor of engagement with health behaviors [32-35]. Many participants—particularly health professionals—highlighted the need for technological interventions to be offered as an adjunct to existing resources. They also stressed the importance of face-to-face communication with care providers to ensure patient understanding of treatment instructions and maintenance of ongoing relationships between patients and health care professionals. This is supported by previous literature, with authors suggesting that technology-delivered health care may be suitable to provide support between face-to-face appointments [24]. It was also noted that patients respond differently to different modes of treatment delivery, with some preferring in-person communication and others preferring online modes. Health professionals were also most likely to have concerns relating to the opportunity cost of the intervention (eg, time taken to learn the technology and teach patients). This fits with recent literature, which states that health professionals are reported to be time-poor and overburdened [36].

Concerns were raised among all 3 groups of participants surrounding the ethicality of the intervention (ie, how well the intervention fit with the participants’ values), but this was mostly discussed by health professionals. Privacy and accuracy of messaging were identified as potential issues, as well as the accessibility of technological interventions for all socioeconomic groups. Issues surrounding privacy and mobile technologies in health care have already been identified in prior research, with some participants expressing concerns about the safety of medical data collected or transmitted via mobile devices [37,38]. Accessibility issues are also of legitimate concern; while 9/10 Australians own a smartphone, rates of use are still low in some groups, including those with disabilities and low household income [39]. Additional research is warranted to explore the potential effects of this disparity.

Interestingly, multiple participants—most commonly health professionals and parents of adolescents with asthma—reported being disappointed with the holographic technology due to high expectations based on the portrayal of holographic technology in movies and TV shows. These preconceived perceptions may have introduced bias in the assessment of this type of technology. Future research with a larger budget may consider upscaling this type of resource to meet preconceived perceptions. Participants also expressed concerns about the burden of using holographic technology more so than VR or AR. The feedback comparing the mixed reality technology modalities led us to amend protocols for future studies limiting intervention content to AR.

### Limitations

The sample of participants was all recruited from 1 hospital in Adelaide, South Australia, limiting the generalizability of the results to the larger population. While smaller than originally planned, this sample size was considered appropriate for the project timeline. Sim et al [40] included such “rules of thumb” as “between 12 and 20 participants in interview studies,” “2 to 10 participants in order to achieve redundancy or saturation,” and “at least five 1-hour interviews for theoretical saturation in grounded theory studies.” Furthermore, recent qualitative studies report similar sample sizes [41-45].

### Implications for Practice

Findings from this study demonstrate that mixed reality resources may be an acceptable treatment/intervention delivery mechanism for young people with asthma. Participants reported feeling positive about the technology and the potential efficacy of this delivery mechanism, and the technology was largely considered easy to use. Health care services might consider the use of mixed reality technology in conjunction with existing resources to diversify treatment delivery and increase engagement.

### Conclusion

The results of this study suggest that mixed reality resources may be an acceptable addition to current health care practices for the purpose of delivering psychological interventions to young people with asthma. Participants were mostly positive about the mixed reality resources; however, some concerns were raised regarding the ethicality, particularly in relation to privacy, accessibility, and accuracy of messaging. Participants noted the need for technology to be used in conjunction with face-to-face engagement with health professionals and noted that some patients would respond to this type of delivery mechanism better than others. Further randomized trials are warranted to explore the effect of mixed reality resources on health and behavior outcomes in this population.

## Appendix

Multimedia Appendix 1 Moderator guide for interviews with young people with asthma.

Multimedia Appendix 2 Moderator guide for interviews with parents of young people with asthma.

Multimedia Appendix 3 Moderator guide for interviews with health professionals.

Multimedia Appendix 4 Questionnaire for young people with asthma.

Multimedia Appendix 5 Questionnaire for parents of young people with asthma.

Multimedia Appendix 6 Questionnaire for health professionals.

Multimedia Appendix 7 Supporting quotes from interviews.

## Abbreviations

AR: augmented reality
CBT: cognitive and behavioral therapy
K10+: Kessler Psychological Distress Scale
TDF: Theoretical Domains Framework
TFA: Theoretical Framework of Acceptability
VR: virtual reality
