# Supplementary material for: Mixed Reality Technology to Deliver Psychological Interventions to Adolescents With Asthma: Qualitative Study Using the Theoretical Framework of Acceptability
Source: JMIR Hum Factors. 2023 Jul 26;10:e34629. doi: 10.2196/34629 (PMC10413228; doi:10.2196/34629)
Supplement: Multimedia Appendix 2 [file humanfactors_v10i1e34629_app2.docx]

## ****Multimedia Appendix 2.** Moderator guide for interviews with parents of young people with asthma.**

## MODERATORS:

Kelsey Sharrad

Kristin Carson-Chahhoud / Zoe Kopsaftis

## LOCATION:

TBA

### **WELCOME:**

### Welcome/who we are:

Welcome. My name in Kelsey Sharrad I am a researcher from The University of South Australia and this is [Kristin Carson-Chahhoud who is an Associate Professor and research fellow] [Zoe Kopsaftis who is a post-doctoral researcher], and we are studying mixed reality technology for the treatment of symptoms of anxiety and/or depression in young people with asthma.

### Why you:

You have been invited to this session because you have identified that your child experiences or has experienced symptoms of anxiety or depression. Our team is trying to determine whether technology such as virtual reality, augmented reality and holographic technology might be useful in delivering cognitive and behavioural therapies to treat these symptoms. To get this information, we need to understand your experience relating to asthma and mental health issues, and need your honest opinions about the mixed reality tools and how they might be useful to your child. We’ll talk briefly about your child’s experience with asthma and symptoms of anxiety and/or depression, but mostly we will discuss your experience with and opinions of the mixed reality tools.

### Honesty/audio taping:

It is very important that we get your honest opinions about the issues and topics during the interview. Remember, there are no wrong answers to what we’ll be talking about. We are interested in your experience and opinions, but everything discussed here will remain completely anonymous. We will be audio recording the session for transcription purposes, however only the direct research team will have access to these audiotapes. You will not be individually identified in any of our presentations or publications.

### Housekeeping:

If you need to take a break at any stage for any reason, please feel free. Let’s begin.

**Some generic probes**

You mentioned __________, tell me more about that.

You mentioned __________, what was that like for you?

You talked about ___________, describe that experience in as much detail as possible.

What else happened?

What were your feelings about that?

It sounds as though you had a pretty strong reaction.

It sounds like you’re saying…….

## INTERVIEW QUESTIONS

**The experience of asthma and mental health issues:**

1. What is your understanding of the relationship between asthma and anxiety or depression?
2. What do you think is a good treatment option for anxiety and depression? Could you give me some examples?
3. Where do you and people your age look for information about mental health and/or asthma? (Why?)
4. What do you think are the barriers to treatment in people your age?

### Cognitive and behavioural therapies:

1. Have you heard of cognitive and behavioural therapies, or CBT, prior to this study? If so, what do you think it is?
2. Do you think CBT is a good tool for treating anxiety and depression in young people? Why or why not?

### Broad technology questions:

1. Does your child use or have access to a smartphone?
2. Do you know if they use any apps relating to health – including for asthma or mental health? What do you think about those sorts of apps?
3. Have you heard of augmented reality, virtual reality, or holographic technologies? What is your understanding of them?
4. Do you think that these technologies would be difficult to use?

### Break to allow participant to use mixed reality tools 15 minutes

### Augmented reality tools:

1. Was it what you expected?
2. What did you like and dislike about the AR tools?
3. What did you think of the design of the tools?
4. Did you find the tools easy to use, or more difficult?
5. How could we make the AR tools better or more engaging?

### Virtual reality tools:

1. Was it what you expected? Why or why not?
2. What did you like and dislike about the VR tools?
3. What did you think of the design of the tools?
4. Did you find the tools easy or difficult to use? Why?
5. How could we make the VR tools better or more engaging?

### Holographic tools:

1. Was it what you expected?
2. What did you like and dislike about the holographic tools?
3. What did you think of the design of the tools?
4. Did you find the tools easy or difficult to use?
5. How could we make the holographic tools better or more engaging?

**Mixed reality tools in general:**

1. What age group do you think these resources were aimed at? Why?
2. Do you think people in this age group would benefit from these tools? Why or why not?
3. Would you, personally, use the mixed reality tools?
4. Are there any other technology-based tools that we should be considering?

### Closing comments:

What else would you like to tell us about your experience with asthma and anxiety, CBT, or about the mixed reality tools? Please tell us about anything else you feel is important for us to know.

Thank you for your time.
