# Supplementary material for: Mixed Reality Technology to Deliver Psychological Interventions to Adolescents With Asthma: Qualitative Study Using the Theoretical Framework of Acceptability
Source: JMIR Hum Factors. 2023 Jul 26;10:e34629. doi: 10.2196/34629 (PMC10413228; doi:10.2196/34629)
Supplement: Multimedia Appendix 3 [file humanfactors_v10i1e34629_app3.docx]

### ****Multimedia Appendix 3.** Moderator guide for interviews with health professionals.**

### **INTRODUCTION:**

### Welcome/what we are trying to achieve

Thank you for agreeing to do this interview. As you know, we are undertaking this research to investigate the potential for mixed reality technology to deliver cognitive and behavioural therapies (CBT) to young people with asthma, for the treatment of symptoms of anxiety and depression. We are conducting these interviews to determine facilitators and barriers of each tool as well as evaluate perceptions of usability, and appropriateness to reach the target audience. We will talk briefly about your experience treating asthma and mental health issues in young people, then you will have an opportunity to trial the tools for yourself, after which time we will discuss your opinions on the tools.

**Honesty/audio taping**

It is very important that we get your honest opinions and the issues and topics during the interview and remember that everything discussed in this interview and your specific opinions will remain completely anonymous. We will be audio recording the session for transcription purposes, however only the direct research team will have access to these audiotapes. You will not be individually identified in any of our presentations or publications.

**Some generic probes**

You mentioned __________, tell me more about that.

You mentioned __________, what was that like for you?

You talked about ___________, describe that experience in as much detail as possible.

What else happened?

What were your feelings about that?

It sounds as though you had a pretty strong reaction.

It sounds like you’re saying…….

## INTERVIEW QUESTIONS

### Asthma and mental health issues:

1. In your experience, how often do you treat a young person with co-morbid asthma and mental health issues?
2. What is your understanding of the relationship between asthma and anxiety or depression?
3. Do you feel that young people feel comfortable speaking with health professionals about mental health issues?
4. Do you feel that you received the necessary training and information to manage symptoms of anxiety and/or depression in young people with asthma?

**Cognitive and behavioural therapies:**

1. What do you recommend as a treatment option for a young person who has asthma with symptoms of anxiety and/or depression?

**Healthcare system:**

1. How do you feel about the way that mental health issues in youth are currently handled in the healthcare system?
2. How do you feel about the role that technology currently plays in healthcare?
3. How would you feel about technology delivering healthcare information and treatment? Could you see any issues arising from this?
4. Do you think that apps relating to health could be useful?

**Novel technology:**

1. What is your understanding of augmented reality, virtual reality, and holographic technologies?
2. Do you think that these technologies would be difficult to use?
3. Do you think that using this kind of technology to deliver CBT would be a good use of your time?

### Break to allow participants to use mixed reality tools 15 minutes

**Augmented reality tools:**

1. Was it what you expected?
2. What did you like and dislike about the AR tools?
3. What did you think about the design of the tools?
4. Did you find the tools easy to use, or difficult?
5. How could we make the AR tools better or more engaging?

**Virtual reality tools:**

1. Was it what you expected?
2. What did you like and dislike about the VR tools?
3. What did you think about the design of the tools?
4. Did you find the tools easy to use, or difficult?
5. How could we make the VR tools better or more engaging?

**Holographic tools:**

1. Was it what you expected?
2. What did you like and dislike about the holographic tools?
3. What did you think about the design of the tools?
4. Did you find the tools easy to use, or difficult?
5. How could we make the holographic tools better or more engaging?

**Smartphone systems:**

1. What age group do you think these resources were aimed at? Why?
2. Do you think people in this age group would benefit from these tools? Why or why not?
3. Do you think learning to use and recommend these resources would be a good use of your time? Why or why not?
4. Do you think young people would respond well to treatment delivered via smartphone?
5. What did you think about the included content? Do you have any comments regarding accuracy and messaging?

### Closing comments

Is there anything else you would like to tell us about asthma, mental health, technology, or the tools? Please tell us about anything else you feel is important for us to know.

Thank you for your time.
