# Supplementary material for: Mixed Reality Technology to Deliver Psychological Interventions to Adolescents With Asthma: Qualitative Study Using the Theoretical Framework of Acceptability
Source: JMIR Hum Factors. 2023 Jul 26;10:e34629. doi: 10.2196/34629 (PMC10413228; doi:10.2196/34629)
Supplement: Multimedia Appendix 5 [file humanfactors_v10i1e34629_app5.docx]

**Multimedia Appendix 5.** Questionnaire for parents of young people with asthma.

**BACKGROUND**

**1. Relationship to child with asthma: ___________________________________________________**

**2. Gender: Male / Female 3. Age: _____________________________**

**4. Nationality? _______________________________________________________________________**

**5. Highest level of formal education:_____________________________________________________**

**5. What area do you live in? (e.g., Western suburbs of Adelaide) _____________________________**

**6. How long have you lived in this location? _______________________________________________**

**7. Could you please list any health problems you have:**

| **Started** | **Health problems** | **Finished** | **On Medication** |
| --- | --- | --- | --- |
| March 2014 | e.g. Wheeze, dry cough, diabetes | Ongoing | Yes – Puffer |
|  |  |  |  |
|  |  |  |  |
|  |  |  |  |
|  |  |  |  |
|  |  |  |  |

**ASTHMA HISTORY**

1. **How old was your child when they were first diagnosed with asthma? _____________________**
2. **Does your child take medications for asthma? Y / N**
3. **If yes, which medication(/s) do they use? __________________________________________________________________________________________________________________________________________________________________**
4. **Has your child ever been hospitalised due to asthma? ___________________________________**
5. **Does anyone in the child’s immediate family have asthma, if so who?______________________**
6. **Please tick what triggers the child’s asthma (tick any that apply and/or add your own)**

|  | Outdoor allergens (pollen, grass etc) |  | Indoor allergens (dust, pet hair etc) |
| --- | --- | --- | --- |
|  | Exercise |  | Household cleaning products |
|  | Stress |  | Foods |
|  | Weather conditions (cold air, humidity) |  | Colds and flus |
|  | Smoke (cigarettes, fires etc) |  |  |
|  |  |  |  |
|  |  |  |  |

**ANXIETY HISTORY**

**1. Does the child experiences any symptoms of anxiety? Y / N**

**2. If yes, what symptoms of anxiety have they experienced? ________________________________**

**____________________________________________________________________________________**

**3. Have you or your child spoken with a health professional about their symptoms of anxiety, if so what type of health professional? __________________________________________________________________**

**4. What do you think triggers your child’s symptoms of anxiety?____________________________**

**____________________________________________________________________________________**

**5. What treatments for anxiety have you heard of? Where did you hear about them? ___________**

**____________________________________________________________________________________**

**____________________________________________________________________________________**

**DEPRESSION HISTORY**

**1. Does your child experiences any symptoms of depression? Y / N**

**2. If yes, what symptoms of depression have they experienced? ______________________________**

**____________________________________________________________________________________**

**3. Have you or your child spoken with a health professional about their symptoms of depression, if so what type of health professional? ____________________________________________________________**

**4. What do you think triggers your child’s symptoms of depression?_________________________**

**____________________________________________________________________________________**

**5. What treatments for depression have you heard of? Where did you hear about them? ___________**

**____________________________________________________________________________________**

**____________________________________________________________________________________**

**HEALTH SCALE QUESTIONNAIRE**

*Please read the following questions and circle the answer that best represents you.*

|  | 1 = strongly agree, 2 = agree, 3 = more or less agree, 4 = undecided,  5 = more or less disagree, 6 = disagree, 7 = strongly disagree |
| --- | --- |
| 1. I know very little about anxiety and depression | (1) (2) (3) (4) (5) (6) (7) |
| 1. I believe that young people find new technology easy to use | (1) (2) (3) (4) (5) (6) (7) |
| 1. Young people prefer to find information about health online, rather than asking their doctor | (1) (2) (3) (4) (5) (6) (7) |
| 1. I feel confident in my child’s ability to manage their asthma | (1) (2) (3) (4) (5) (6) (7) |
| 1. Cognitive and behavioural therapies (CBT) is a useful treatment for symptoms of anxiety and/or depression | (1) (2) (3) (4) (5) (6) (7) |
| 1. I think symptoms of anxiety and/or depression are common in young people | (1) (2) (3) (4) (5) (6) (7) |
| 1. I am familiar with novel technologies such as augmented reality, virtual reality, and holographic technology | (1) (2) (3) (4) (5) (6) (7) |
| 1. I believe my child would benefit from some form of treatment for their symptoms of anxiety and/or depression | (1) (2) (3) (4) (5) (6) (7) |
| 1. Technology, such as smartphone apps can be a good educational tool for young people | (1) (2) (3) (4) (5) (6) (7) |
| 1. Asthma contributes to my child’s symptoms of anxiety and/or depression | (1) (2) (3) (4) (5) (6) (7) |

**SOFTWARE REVIEW**

*Please read the following questions and circle the answer that best represents you.*

| How easy is it to navigate (move from one feature to another) through the tools? | Very  poor | Poor | Fair | Good | Very  good |
| --- | --- | --- | --- | --- | --- |
| How easy is it to **learn** how to use the tools and features? | Very  poor | Poor | Fair | Good | Very good |
| How easy is it to **use** the tools and features? | Very  poor | Poor | Fair | Good | Very good |
| How attractive is visual design (fonts and colours)? | Not attractive at all | Not attractive | Fair | Attractive | Very attractive |
| Do the tools appear well-organised? | Very  poor | Poor | Fair | Good | Very good |
| Are the sizes of the fonts/buttons/videos appropriate? | Very  poor | Poor | Fair | Good | Very good |
| Is the content presentation interesting? | Very  poor | Poor | Fair | Good | Very good |
| What did you think about the quality of the tools? | Very  poor | Poor | Fair | Good | Very good |
| Are the tools irritating? | Very  poor | Poor | Fair | Good | Very good |
| How easy is it to customise the content to your needs? | Very  poor | Poor | Fair | Good | Very good |
| How interesting are the tools? | Very  poor | Poor | Fair | Good | Very good |
| What are your thoughts about the accuracy of the information? | Very  poor | Poor | Fair | Good | Very good |
| What do you think about the presentation of the information? | Very  poor | Poor | Fair | Good | Very good |
| How would you rate the sufficiency of the information?  (i.e. clear and concise) | Very  poor | Poor | Fair | Good | Very good |
| Is the intended use and purpose of the tools clear? | Very  poor | Poor | Fair | Good | Very good |

*Scale adapted from Enlight protocol (Baumel 2017).*
