# Supplementary material for: Mixed Reality Technology to Deliver Psychological Interventions to Adolescents With Asthma: Qualitative Study Using the Theoretical Framework of Acceptability
Source: JMIR Hum Factors. 2023 Jul 26;10:e34629. doi: 10.2196/34629 (PMC10413228; doi:10.2196/34629)
Supplement: Multimedia Appendix 6 [file humanfactors_v10i1e34629_app6.doc]

**Multimedia Appendix 6.** Questionnaire for health professionals.

**BACKGROUND**

**1. Qualification: _____________________________________________________________________**

**2. Nationality: _______________________________________________________________________**

**3. Current occupation and employer: ___________________________________________________**

**4. Years with current employer: ________________________________________________________**

**5. Gender: Male / Female 6. Age: ______________________**

**7. How many years of experience do you have in this field? _________________________________**

**8. Do you have any personal experience with asthma? _____________________________________**

**ASTHMA AND MENTAL HEALTH**

1. **Do you have experience treating young people with asthma? Y / N**
2. **What are some of the symptoms of anxiety ? 3. What are some of the symptoms of depression?**

**________________________________________________________________________________________________________________________________________________________________**

1. **Research suggests that half of young people with asthma also experience symptoms of anxiety and/or depression. Has this been your experience? Elaborate if possible.**

**__________________________________________________________________________________________________________________________________________________________________**

**_________________________________________________________________________________**

1. **What do you currently advise for young patients with asthma who present with symptoms of anxiety and/or depression?**

**__________________________________________________________________________________________________________________________________________________________________**

1. **Do you find CBT to be an effective tool for treating symptoms of anxiety and/or depression in young people? Why/Why not?**

**__________________________________________________________________________________________________________________________________________________________________**

**HEALTH SCALE QUESTIONNAIRE**

*Please read the following questions and circle the answer that best represents you.*

|  | 1 = strongly agree, 2 = agree, 3 = more or less agree, 4 = undecided,  5 = more or less disagree, 6 = disagree, 7 = strongly disagree |
| --- | --- |
| 1. I am very confident that I could manage a patient with asthma and symptoms of anxiety/depression on my own | (1) (2) (3) (4) (5) (6) (7) |
| 1. I have the knowledge and skills necessary to help a young person manage their symptoms of anxiety/depression | (1) (2) (3) (4) (5) (6) (7) |
| 1. I believe symptoms of anxiety are managed poorly in current practice | (1) (2) (3) (4) (5) (6) (7) |
| 1. I believe symptoms of anxiety and depression are easily recognised among young people with asthma | (1) (2) (3) (4) (5) (6) (7) |
| 1. I believe symptoms of anxiety and depression are managed well by health professionals for young people with asthma | (1) (2) (3) (4) (5) (6) (7) |
| 1. I feel that technology would be an effective medium to engage young people in education | (1) (2) (3) (4) (5) (6) (7) |
| 1. I think CBT would be very effective if delivered via technology for young people with symptoms of anxiety and/or depression | (1) (2) (3) (4) (5) (6) (7) |
| 1. Symptoms of anxiety and/or depression are common in young people with asthma | (1) (2) (3) (4) (5) (6) (7) |
| 1. CBT delivered via technology to treat symptoms of anxiety and/or depression in young people with asthma would make my job easier | (1) (2) (3) (4) (5) (6) (7) |
| 1. Young people do not tend to seek help from health professionals for symptoms of anxiety and depression | (1) (2) (3) (4) (5) (6) (7) |

**SOFTWARE REVIEW**

*Please read the following questions and circle the answer that best represents you.*

| How easy is it to navigate (move from one feature to another) through the tools? | Very  poor | Poor | Fair | Good | Very  good |
| --- | --- | --- | --- | --- | --- |
| How easy is it to **learn** how to use the tools and features? | Very  poor | Poor | Fair | Good | Very good |
| How easy is it to **use** the tools and features? | Very  poor | Poor | Fair | Good | Very good |
| How attractive is visual design (fonts and colours)? | Not attractive at all | Not attractive | Fair | Attractive | Very attractive |
| Do the tools appear well-organised? | Very  poor | Poor | Fair | Good | Very good |
| Are the sizes of the fonts/buttons/videos appropriate? | Very  poor | Poor | Fair | Good | Very good |
| Is the content presentation interesting? | Very  poor | Poor | Fair | Good | Very good |
| What did you think about the quality of the tools? | Very  poor | Poor | Fair | Good | Very good |
| Are the tools irritating? | Very  poor | Poor | Fair | Good | Very good |
| How easy is it to customise the content to your needs? | Very  poor | Poor | Fair | Good | Very good |
| How interesting are the tools? | Very  poor | Poor | Fair | Good | Very good |
| What are your thoughts about the accuracy of the information? | Very  poor | Poor | Fair | Good | Very good |
| What do you think about the presentation of the information? | Very  poor | Poor | Fair | Good | Very good |
| How would you rate the sufficiency of the information?  (i.e. clear and concise) | Very  poor | Poor | Fair | Good | Very good |
| Is the intended use and purpose of the tools clear? | Very  poor | Poor | Fair | Good | Very good |

*Scale adapted from Enlight protocol (Baumel 2017).*
