# Supplementary material for: Mixed Reality Technology to Deliver Psychological Interventions to Adolescents With Asthma: Qualitative Study Using the Theoretical Framework of Acceptability
Source: JMIR Hum Factors. 2023 Jul 26;10:e34629. doi: 10.2196/34629 (PMC10413228; doi:10.2196/34629)
Supplement: Multimedia Appendix 7 [file humanfactors_v10i1e34629_app7.docx]

**Multimedia Appendix 7.** Supporting quotes from interviews.

| TFA Construct | Example quotes | Participant |
| --- | --- | --- |
| Anticipated affective attitude | *“I think certainly novel in a different way of engaging. So that's going to reach different audience or reach the same audience in a slightly different way. So, like I said, it just broadens out your options a little bit sometimes that, just that engagement, "Oh, this is different" people tune in because it's different.”*  *“I think it's really cool thing, especially if you're like going through therapy to help with your fears or something like that. I find that very cool. And, amazing.”*  *“If they were informative and, you know, gave the patient lots of information and alternatives and if they are really well done, then, then that would be great.”* | Health professional  Young person with asthma  Parent of young person with asthma |
| Experienced affective attitude | *“Uh, I, I must admit I had kind of maybe more ridiculous expectations. Like I thought it would be a matter of just like coming up in front of me somehow as opposed to having to look through the pyramid. Yeah.”*  *“I mean an old bloke like me think's its bloody fantastic, so I don't think there's any limit really”*  *“Yes of course I would. These like really work, very informative and very, very calming”* | Health professional (about holographic technology)  Parent of young person with asthma  Young person with asthma, when asked if they would use these tools |
| Anticipated effectiveness | *“Um, it's just another learning tool. But, um, one that would address so many different types of learning types being able to see, do yeah. Um, yeah. Rather than just reading on paper and things like that. Yeah. So many people are visual learners.”*  *“Um, yeah, definitely. It would help. A lot because you know a lot of people spend most of their time on their phones nowadays, having an app like that. Well, you know, it would probably help a lot and you would be more, you know, you would probably use it more.”* | Health professional  Young person with asthma |
| Experienced effectiveness | *“Can't see why they wouldn't benefit any more than any other way of doing it? Again, it comes down to engagement. This is a tool that is bound to be engaging. If the content is good, then it should, should be effective.”*  *“I suppose as an adjunct to other therapies it might, it would be worth trying. Um, yeah. So it might be a different way of trying to get someone to engage in some breathing or relaxation exercises. Um, maybe they're going to be more interested than they might if it was just a audio track.”*  *“Um, not really. I mean, it serves a purpose and does, does the job. Yeah. Um, yeah, it's already bright and colourful and right there, and it's got that 3D aspect and you know, it's a good tool for kids to help understand something and see it visually. So, no, I think it's good.”*  *“Yeah, it definitely would. It would like, you know, if you were in a place that, you know, that was kind of like making you, you know, you know, like very stressed and that. If you put like the headset on that was there, you'd forget that you're there. And like with the meditation, it can also like, help you calm down and then, you know, bring you to a state where you just feel calm and you're ready to go back.”* | Health professional  Health professional  Parent of young person with asthma  Young person with asthma, when asked if delivering interventions via virtual reality would be a good idea |
| Ethicality | *“Uh, it’s got advantages and disadvantages. – But it was a concern because that can be falsely reassuring. And some of these children were, um, uh, at risk or identified as being at risk in terms of neglect and abuse. And sometimes physically seeing a child is, is important”*  *“Um, I think I have concerns over privacy and access to data and so on, in particular as most, um, servers seem to be based overseas. And so the question of what happens to your data is a significant one and probably one that needs to be thought through carefully with some of, some of the things that we, we do, you know, in our line of work.”*  *“I think the only risk is around that. I don't mean this in a bad way, but around compliance and that understanding that if you give the technology intervention and then they go do it and you say, "how was that?" and they go "good". what does, and then trying to unpack. So what did they get out of it? Did they, did they have interaction with it or did they just sort of go through the motions with it? Did they understand if there's language in it? Do they understand the, sort of the language or the concepts behind this? I think the risk is around that sort of meaningful engagement as opposed to just having it.”*  *“And what happens if your internet breaks down?”*  *“I think that it's important that not everything is self-diagnosed and then self referred to sort of technology treatments, I suppose. I think probably having an element of, um, human interaction is important. I think one of the things that doesn't get discussed with, um, sort of online, um, you know, treatment programs for anxiety is just the actual benefit of connection with someone which may be the underlying issue rather than, even though someone might be saying that depressed, connection's a big part of treatment of depression or anxiety as well.”* | Health professional  Health professional  Health professional  Parent of young person with asthma  Health professional |
| Anticipated opportunity costs | *“If it was really quality information, um, and it engaged them more than say, just chatting with me, like if they're really engaged and really taking it in and, um, the information was good, then it would be a really good use of time.”*  *“I would be unlikely to use my time to do this sort of teaching or, or, um, therapy.”* | Health professional  Health professional |
| Experienced opportunity costs | *“Uh, I don't feel like they would be hard to learn how to use, so I don't feel like you'd been losing a lot of other productivity time or anything like that. I think it would have been fairly straightforward to learn how to use them, um, and then potentially become a really good use of your time because while they're watching whatever it is for however long it goes for, you can be, you know, setting up something else for them or working on something else. And they've got that information, you check back in with them to see, you know what they've gotten from it and sort of build from there, I guess.”*  *“The benefits outweigh, you know, you just, it's just, it's something else to learn, but that's fine. It's good benefits to it.”*  *“Oh, that's, I mean, that's very simple. Um, so I think, um, I think you couldn't ask for something to be more accessible than that really. Um, with the link and, um, and, bring up the app and um, and just watch it.”* | Health professional  Parent of young person with asthma  Parent of young person with asthma |
| Anticipated burden | *“Depending on what age you were.”*  *“Um, no, not really. It really depends, like you know how there's like millennials and gen Z and that. I'm not really that known on those things, but yeah, like those people, like they would know a lot about technology, but the older ages, um, yeah, some people do, have learnt to develop like a known thing about technology. So I feel like it would be really easy to use. Yeah.”*  *“I'm assuming it's probably not difficult to use. Yeah. Technology seems to get more, more advanced and it doesn't seem to be that tricky.”* | Parent of young person with asthma  Young person with asthma  Health professional |
| Experienced burden | *“The only thing I thought that it was obviously fiddly. That's obviously something you will work on as you develop it.”*  *“Yeah. I think like [child’s name] and that would probably handle it quite readily and accessible. But for me, the technology side of it, I'd sort of have trouble with.”*  *“Oh, that's, I mean, that's very simple. Um, so I think, um, I think you couldn't ask for something to be more accessible than that really. Um, with the link and, um, and, bring up the app and um, and just watch it.”*  *“Um, it could be, yeah. It could be easy to use. I would call that one of the easier things to you, you know?”* | Health professional, when asked about holographic technology  Parent of young person with asthma  Parent of young person with asthma  Young person with asthma, about augmented reality technology |
| Self-efficacy | *“Um, yes, because if I know how to turn on an app and register information, yes.”*  *“Probably difficult for my age group. Um, because you know, we didn't grow up with them, but the kids it's just like, they don't find it difficult at all.”*  *“I would personally use it. Yeah. If I was unsure about something or if I wanted to learn something, I'm like, I would definitely use this. It's good to have different resources to go to.”* | Health professional, when asked if they would be comfortable using apps/technology  Parent of young person with asthma  Young person with asthma |
| Intervention coherence | *“Well, young people are very visual, so I think they'll really like being able to watch that it's like it came to life. Like it was real, which I think they will like more and will engage them. And there's so much you could use that for”*  *“Well, like if you want to be like more of a bit of an educational side, you could also, like, I guess could have been like a video, video or something of like demonstrating how asthma works and like whatnot. That'd be like really helpful to like looking at both like figuring out like how asthma works and how, you know, help, you know, control it.”*  *“I'm trying to promote it cause there's a lot of, lot of kids that, you know, chronic illnesses that are quite institutionalized, that we do regular things to them that they hate. So why not try and take them off to another place and, you know, make it not as scary for them.”* | Health professional  Young person with asthma  Parent of young person with asthma |

*Key: TFA=Theoretical Framework of Acceptability, gen Z = generation Z,*
